# Supplementary material for: Carbon-Based Ternary Nanocomposite: Bullet Type ZnO–SWCNT–CuO for Substantial Solar-Driven Photocatalytic Decomposition of Aqueous Organic Contaminants
Source: Molecules. 2022 Dec 12;27(24):8812. doi: 10.3390/molecules27248812 (PMC9784868; doi:10.3390/molecules27248812)
Supplement: Supplementary file 1 [file molecules-27-08812-s001.zip › molecules-2021154-supplementary.pdf]

Carbon-based ternary nanocomposite: bullet type ZnO–SWCNT–CuO for substantial solar-driven photocatalytic decomposition of aqueous organic contaminants

Santu Shrestha[a,b], Kamal Prasad Sapkota[a,b], Insup Lee[a], Akherul Islam[c], Anil Pandey[a,b], Narayan Gyawali[a], Jeasmin Akter[a], Harshavardhan Mohan[a], Taeho Shin[a], Sukmin Jeong[d], Jae Ryang Hahn[a,e*]

[a]*Department of Chemistry, Jeonbuk National University, Jeonju 54896, Republic of Korea*
[b]*Department of Chemistry, Tribhuvan University, Amrit Campus, Kathmandu, Nepal*
[c]*Department of Bioactive Material Sciences, Jeonbuk National University, Jeonju 54896, Republic of Korea*
[d]*Department of Physics, Jeonbuk National University, Jeonju 54896, Republic of Korea*
[e]*Textile Engineering, Chemistry and Science, North Carolina State University, 2401 Research Dr., Raleigh, NC 27695, USA*

* Corresponding author

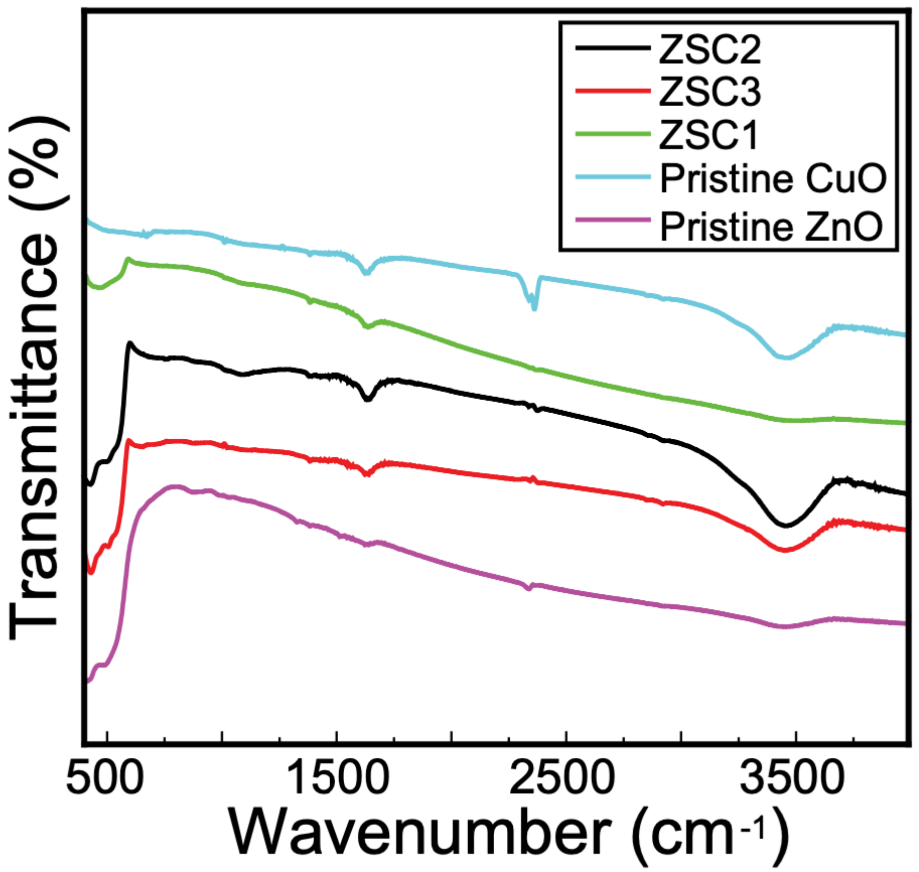

Figure S1. FTIR spectra of ZSC1, ZSC2, ZSC3, pristine CuO, and pristine ZnO.

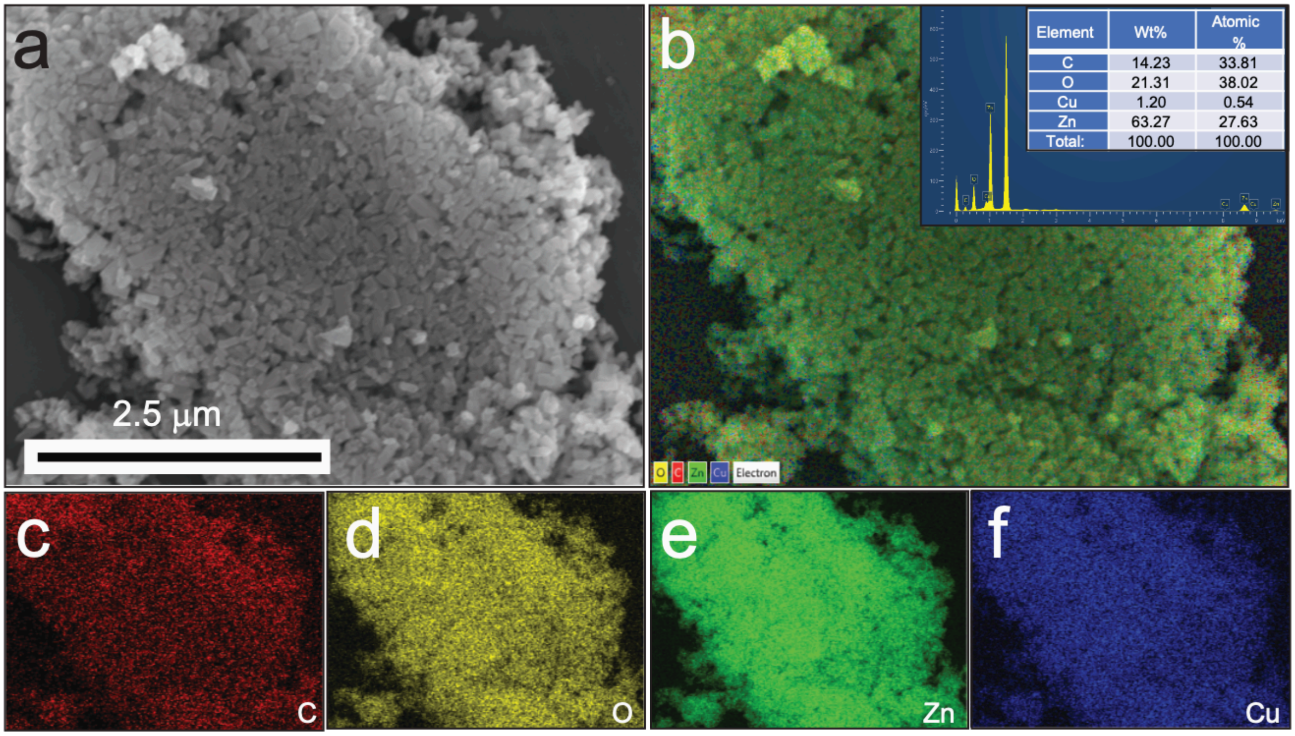

Figure S2. EDS mapping of ZSC3: (a) FE-SEM image; (b) EDS image of (a), where C, O, Zn, and Cu, are represented by red, yellow, green, and blue, respectively. (c–f) Element distribution images of (c) C, (d) O, (e) Zn, and (f) Cu in the nanocomposite.

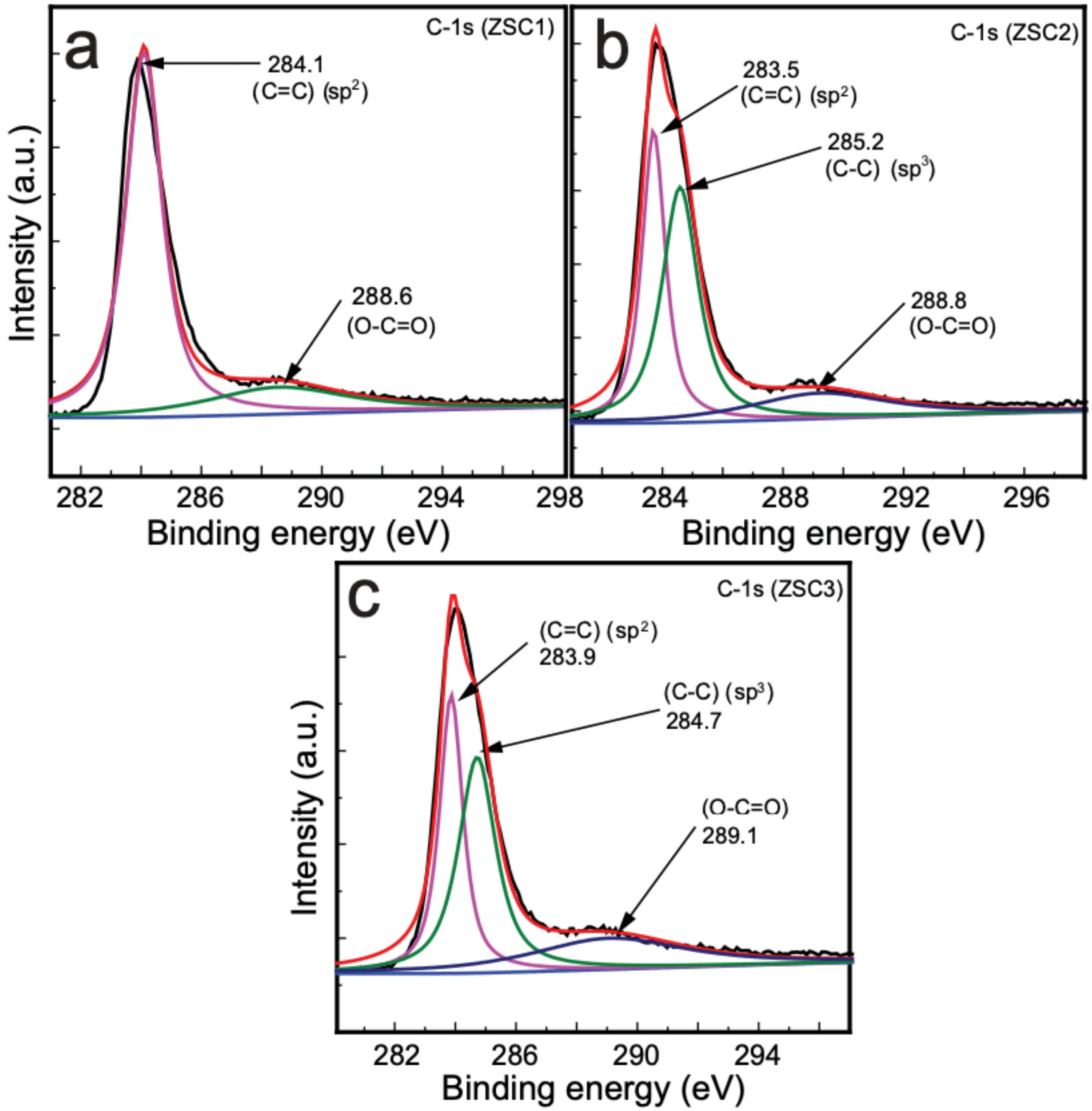

Figure S3. (a) C-1*s* core-level spectra of (a) ZSC1, (b) ZSC2, and (c) ZSC3.

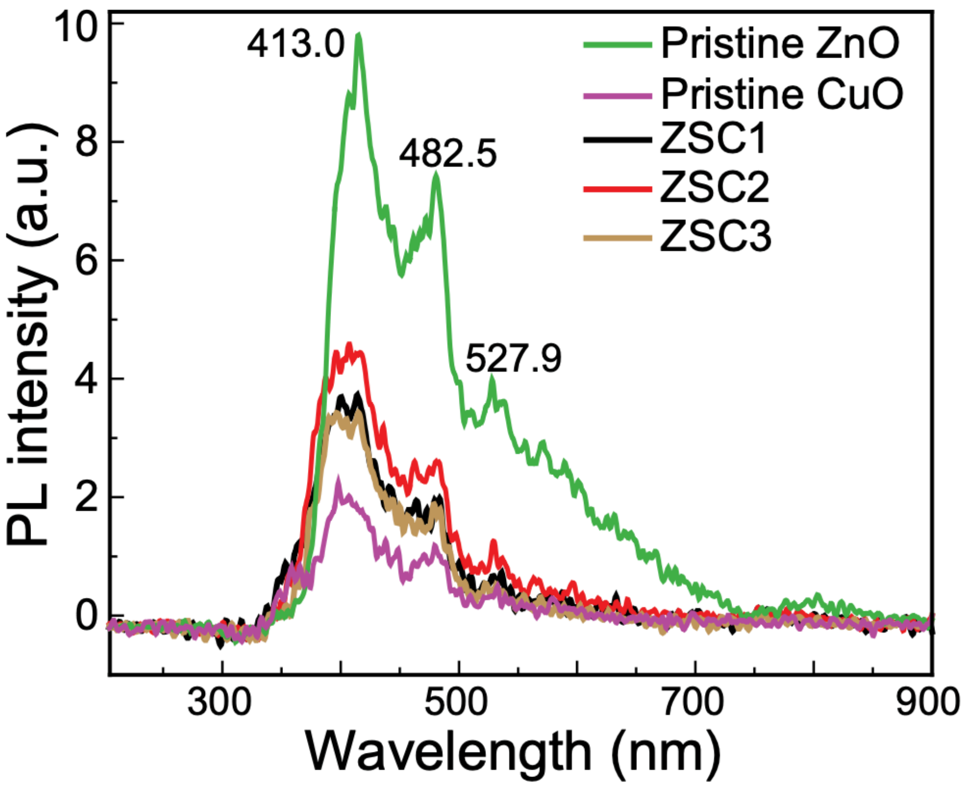

Figure S4. PL spectra of the pristine samples and the composites.

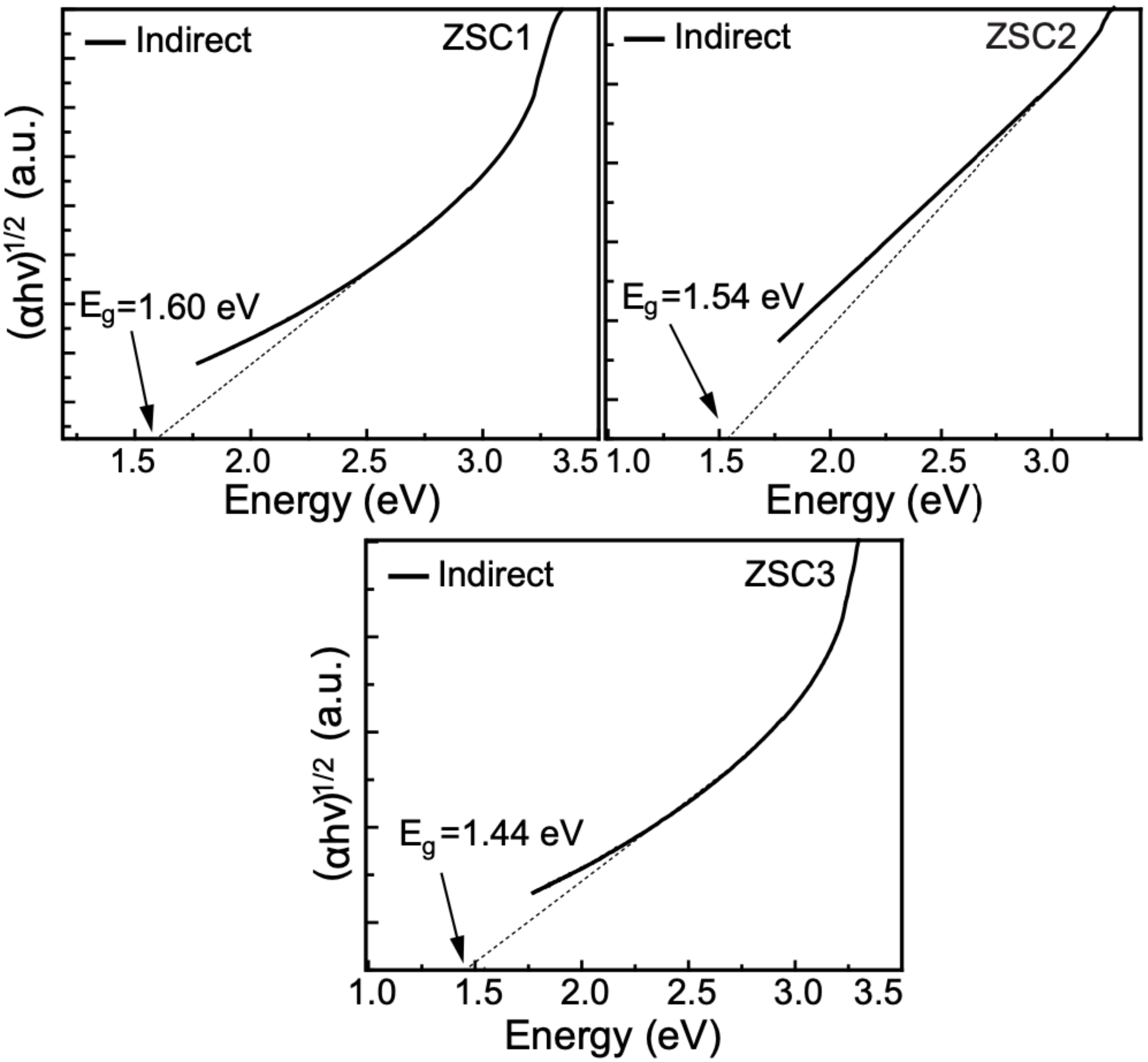

Figure S5. Tauc plots for the ZSC samples, as obtained via the indirect bandgap method.

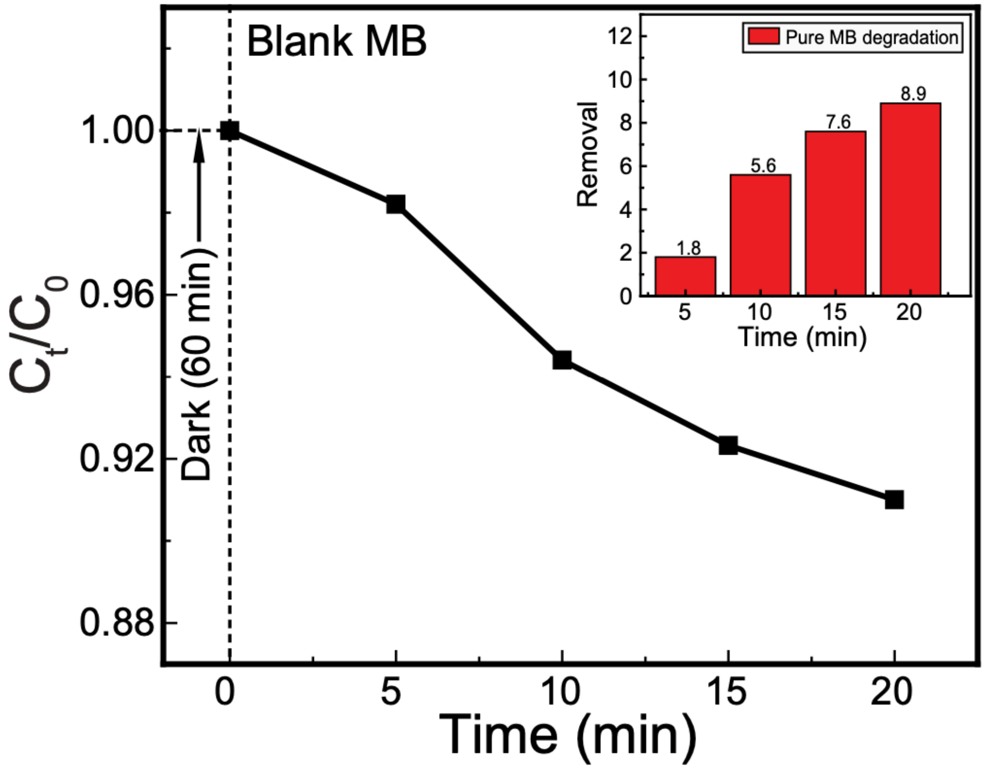

Figure S6. Photodegradation of MB as a function of time in the absence of a photocatalyst.

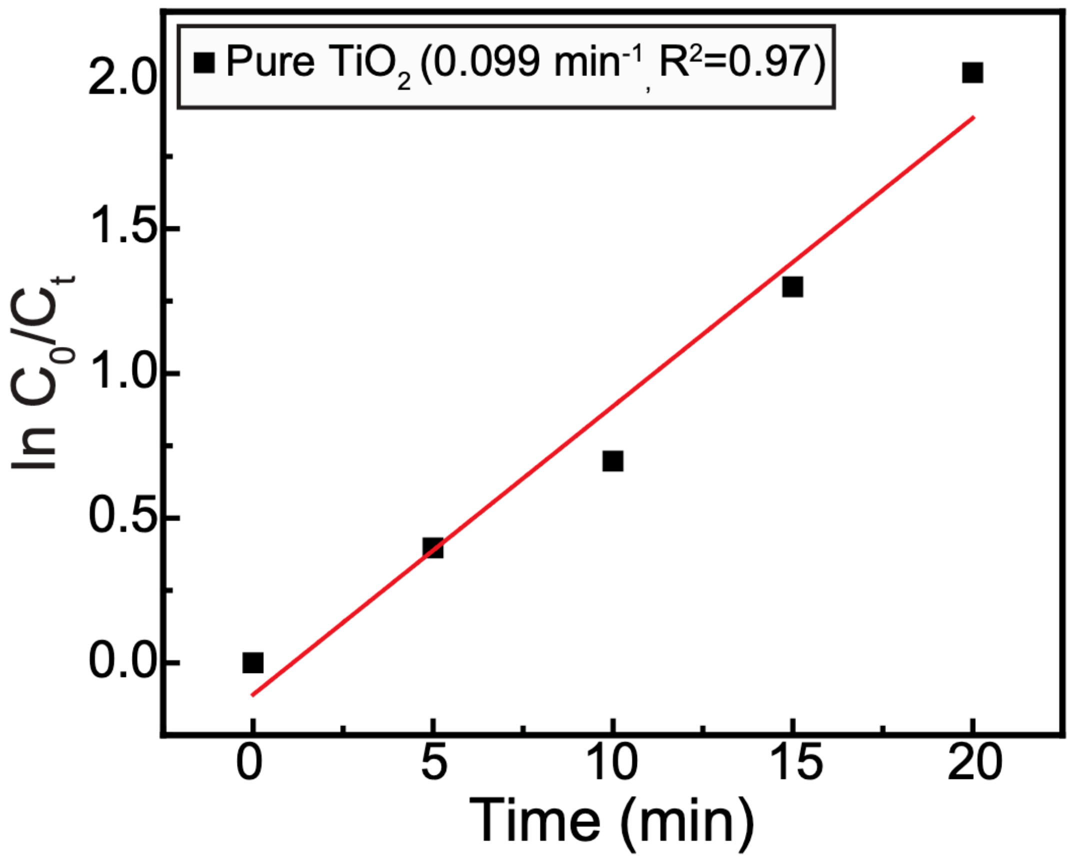

Figure S7. Kinetic plot for MB degradation in the presence of pure TiO_2 (Degussa p25).

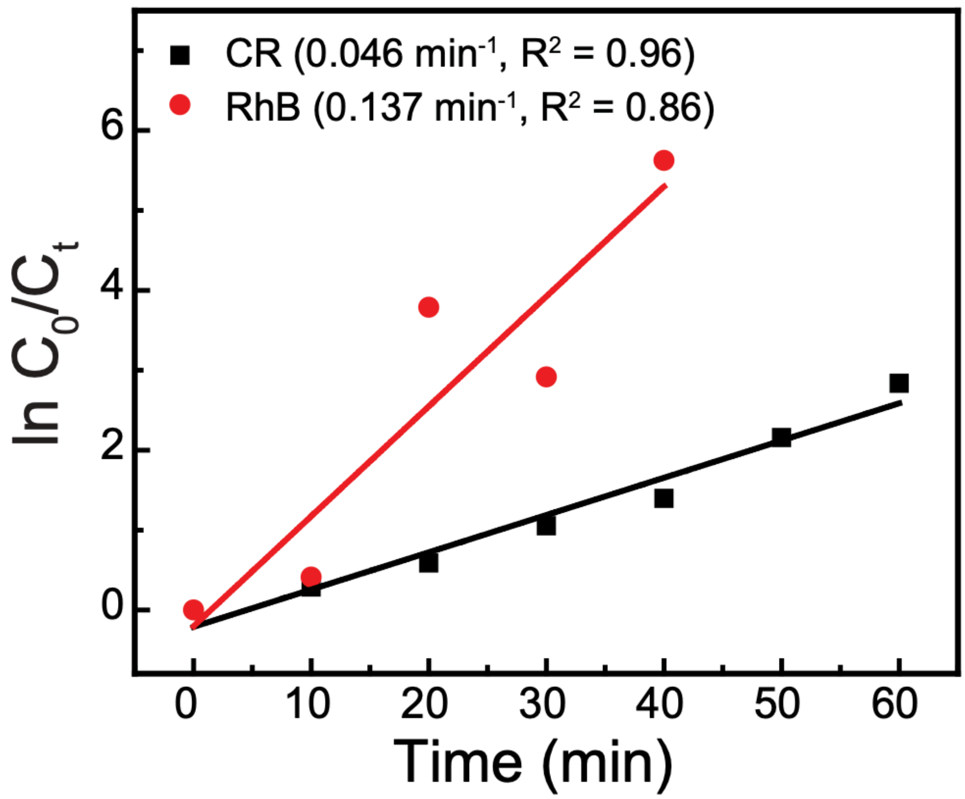

Figure S8. Kinetic plot for photodegradation of CR and RhB in the presence of ZCS3.

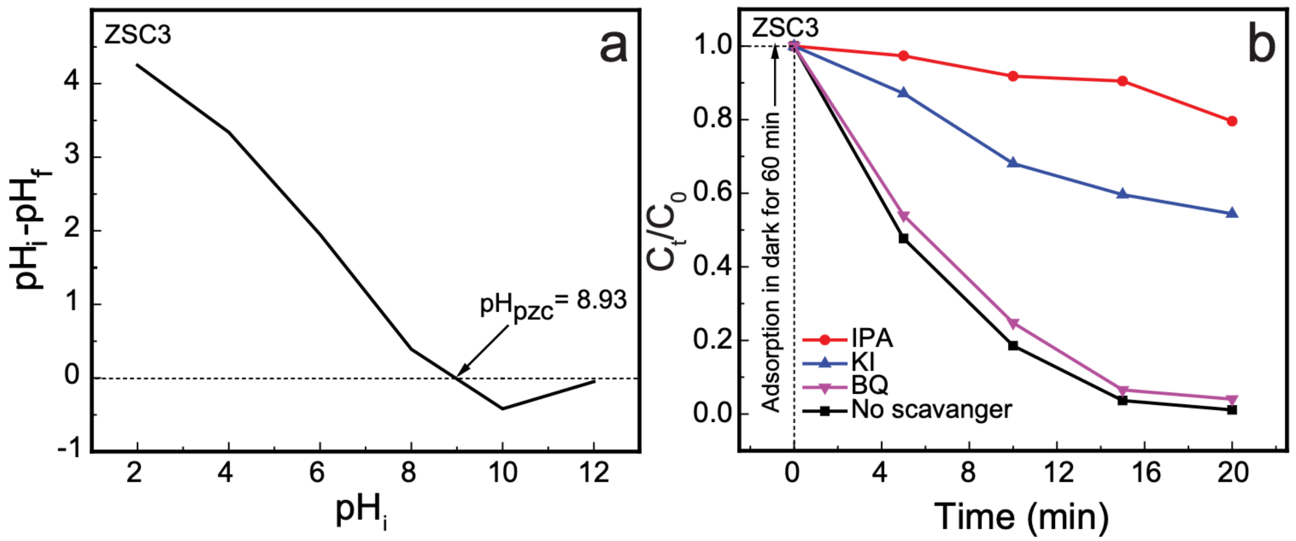

Figure S9. (a) Determination of the point of zero charge of the ZSC3 photocatalyst. (b) Effects of IPA, KI, and BQ charge-carrier scavengers on the photocatalytic decomposition of MB.

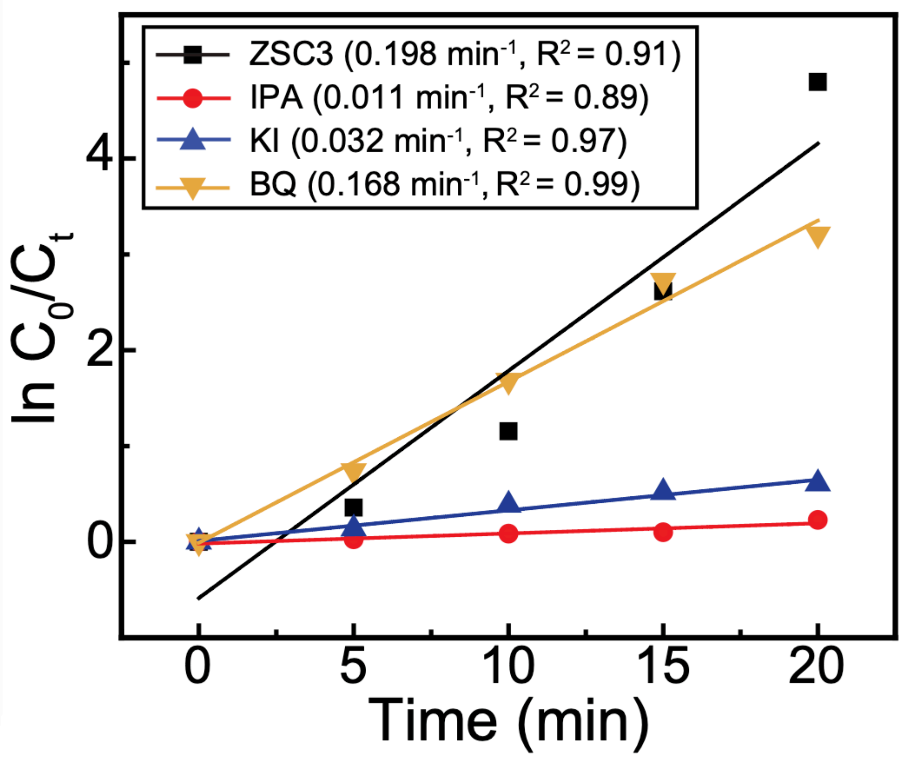

Figure S10. Kinetic plots for active species trapping agents.

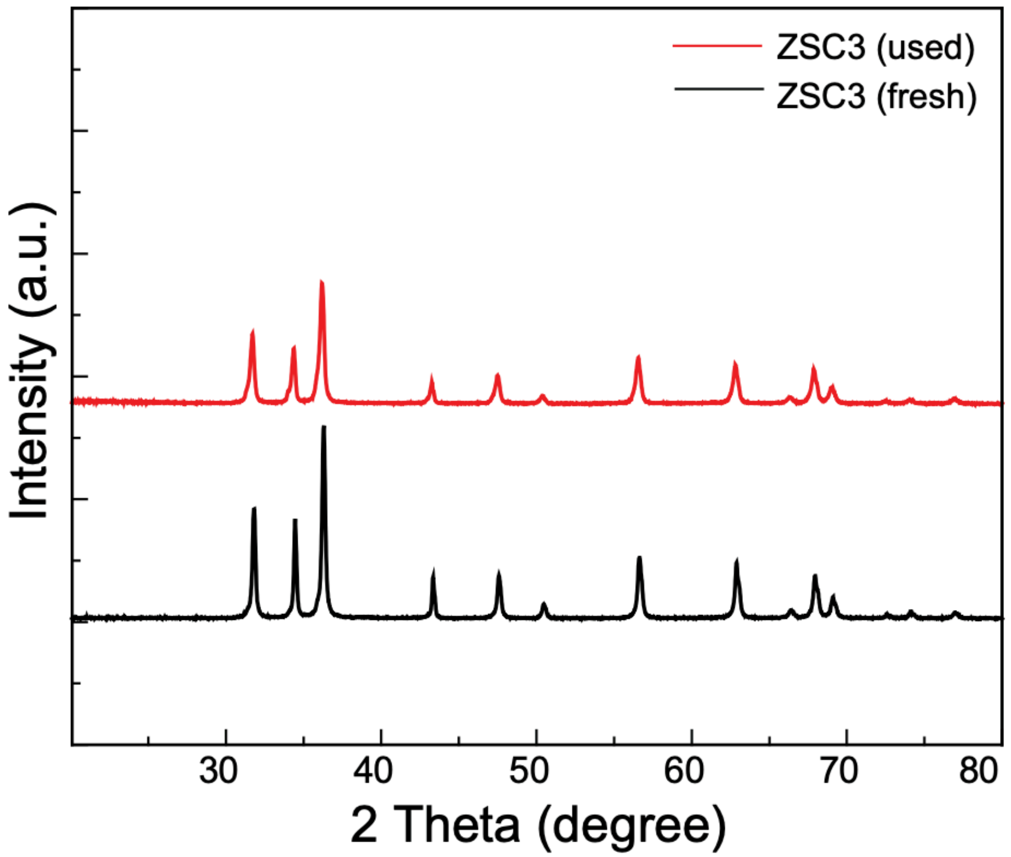

Figure S11. XRD plots of the ZSC3 before and after use four times.

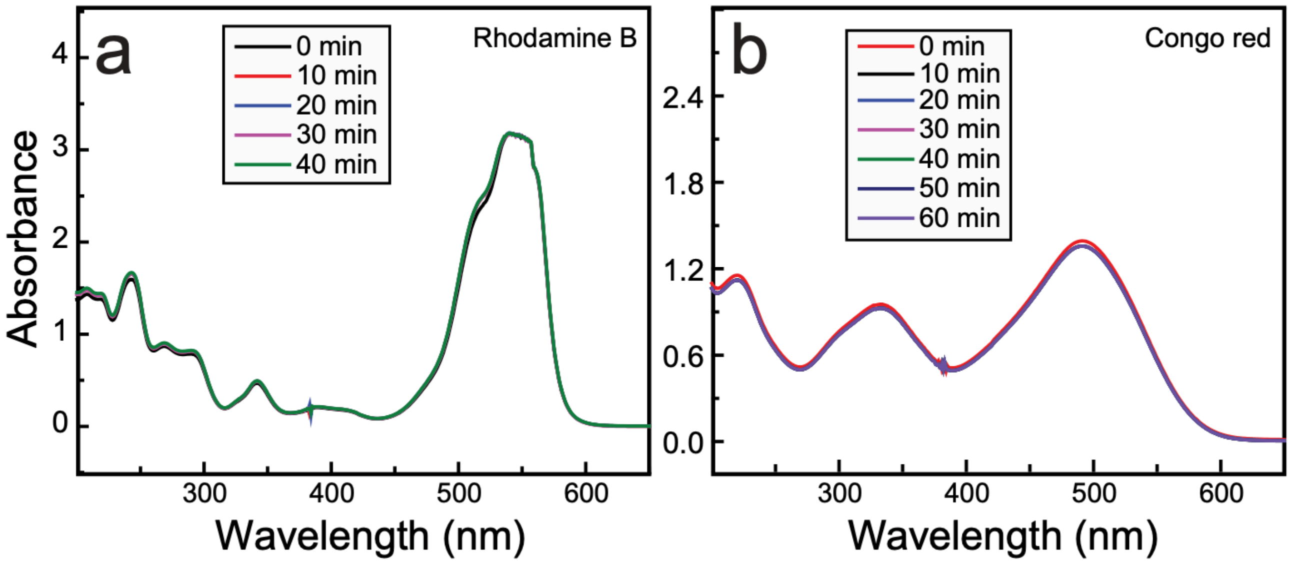

Figure S12. UV-vis spectra of (a) Rhodamine B, and (b) Congo red for the direct photolysis (without photocatalysts).

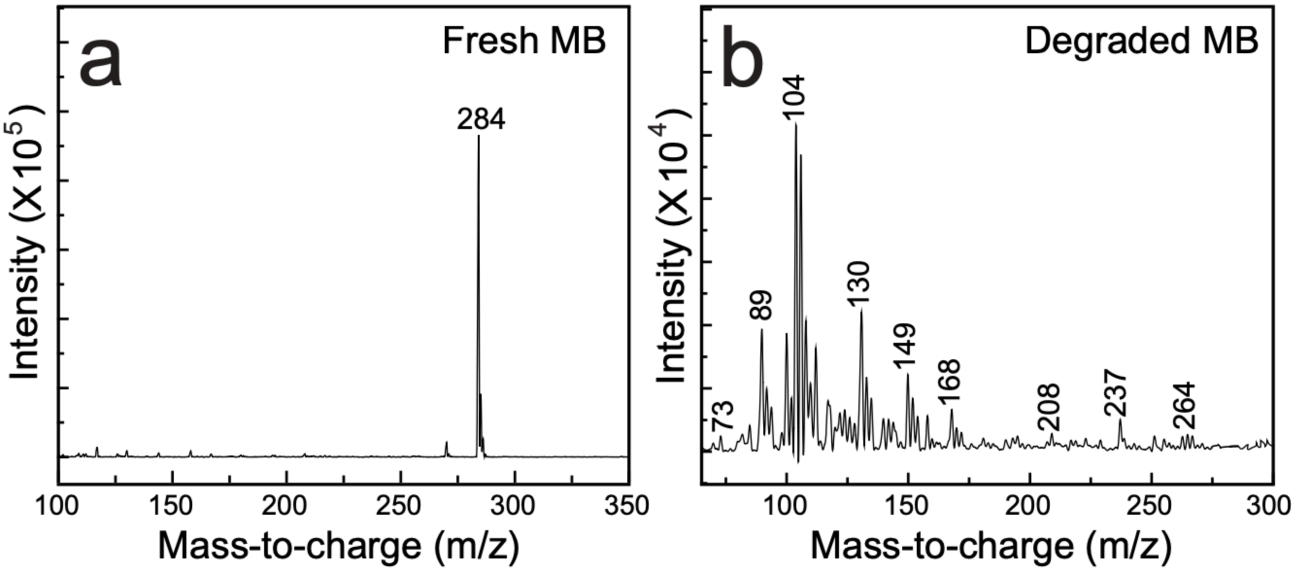

Figure S13. MS data for an MB solution (a) fresh MB, and (b) degraded MB using ZCS3 under simulated sunlight for 20 min.

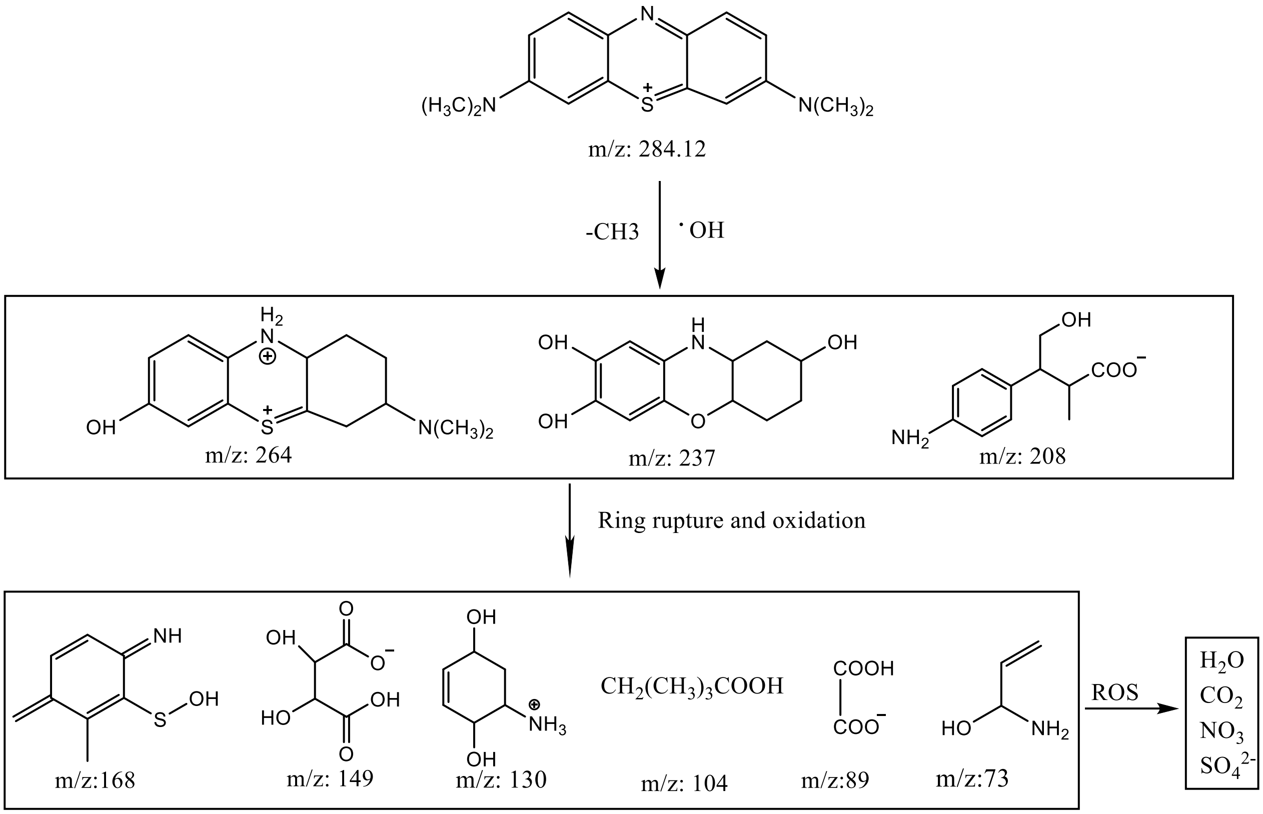

Figure S14. The degradation pathway of MB.

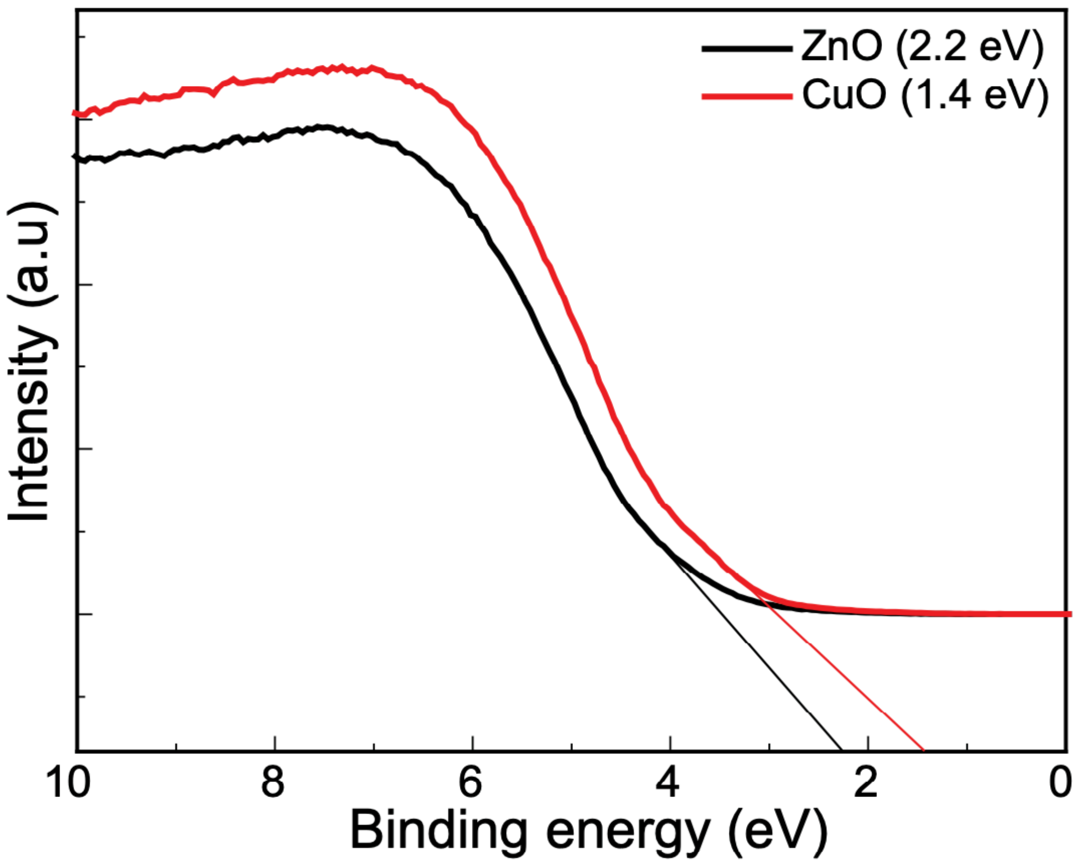

Figure S15. UPS spectra of ZnO and CuO.
